# Recent progress in the application of hydrophilic interaction liquid chromatography for the separation and analysis of traditional Chinese medicine

**DOI:** 10.3389/fchem.2025.1694998

**Published:** 2025-10-01

**Authors:** Yuanyuan Wu, Hui Huang, Xianglong Zhao, Junhao Lin, Jialiang Guo, Zhengjin Jiang, Kesara Ar-Sanork, Tao Deng, Dongsheng Xu

**Affiliations:** 1 College of Pharmacy/State Key Laboratory of Bioactive Molecules and Druggability Assessment/International Cooperative Laboratory of Traditional Chinese Medicine Modernization and Innovative Drug Development of Ministry of Education (MOE) of China, Institute of Pharmaceutical Analysis, Jinan University, Guangzhou, China; 2 School of Medicine, Foshan University, Foshan, China; 3 School of Chemistry, Institute of Science, Suranaree University of Technology, Nakhon Ratchasima, Thailand

**Keywords:** traditional Chinese medicine, hydrophilic interaction liquid chromatography, polar compounds, stationary phases, quality control

## Abstract

Traditional Chinese medicine (TCM), with a history spanning over 2,000 years, contains numerous strongly polar components that are increasingly the focus of pharmaceutical and analytical research. However, conventional reversed-phase liquid chromatography usually fails to adequately retain and separate these highly polar components. Hydrophilic interaction liquid chromatography (HILIC) has emerged as a powerful complementary technique, offering improved retention and separation for polar analytes. In recent years, HILIC has seen rapid development and wide application in the analysis of TCM. This review summarizes the major types of HILIC stationary phases and their recent applications in the separation of polar components in TCM. Particular attention is given to the analysis, quality control, and metabolomic profiling of bioactive compounds such as flavonoids, alkaloids, and saponins, highlighting the increasingly important role of HILIC in promoting TCM research.

## 1 Introduction

In the modernization of traditional Chinese medicine (TCM), highly polar components such as polysaccharides, organic acids, and glycosides have become key research targets in analytical chemistry and biochemistry due to their potential bioactive value. These ingredients not only serve as the material basis for the synergistic effects of TCM compounds, but also exhibit therapeutic potential in key pharmacological pathways, including immunomodulation, anti-inflammatory activity, antioxidant effects, and metabolic regulation (Lin et al., 2019).

Hydrophilic interaction liquid chromatography (HILIC), first pioneered by Alpert (1990), has emerged as an important complementary technique to reversed-phase liquid chromatography (RPLC), enhancing the retention of polar compounds through the use of organic and aqueous mobile phases via hydrophilic interactions, including dipole-dipole (polar interactions), hydrogen bonding, and electrostatic interactions (Sheng et al., 2023). In recent years, interest in HILIC has grown steadily, and it is now widely applied in biomedical research, proteomics, and metabolomics. Its strong retention capacity for polar compounds makes HILIC particularly valuable for the analysis of polar analytes in TCM, overcoming the limitations of traditional RPLC.

**FIGURE 1:**
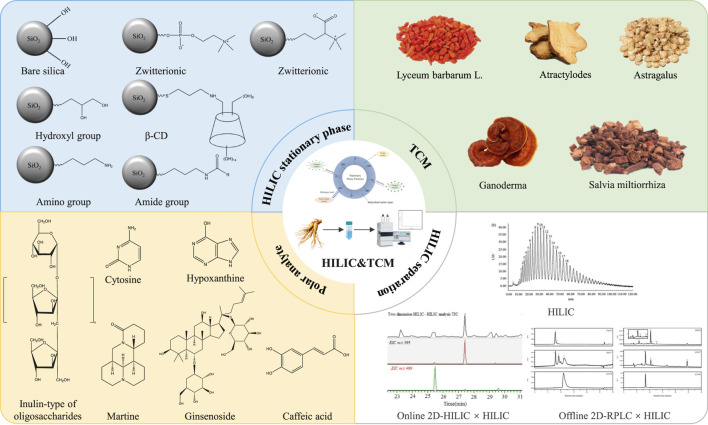
Overview of HILIC stationary phases and their applications for analyzing polar components of TCMs, as presented in this review.

While previous reviews have primarily focused on the general progress of component separation in TCM, the purpose of this paper is to systematically review recent advances in the field, and ultimately to provide a solution for overcoming the challenges of separating polar components in TCM by integrating key research achievements (Figure 1).

## 2 HILIC stationary phase

The separation performance of HILIC is highly dependent on the composition of the stationary and mobile phases (Jandera and Janás, 2017). As the core of HILIC, stationary phases are diverse and exhibit complex mechanisms of action (Guo, 2022). According to the charge classification of stationary phase ligands, they can be divided into neutral, anionic, cationic, and zwitterionic stationary phases.

There are many types of neutral stationary phase ligands, including hydroxyl groups (diols and cyclodextrins), amide groups (amides and urea), and zwitterionic stationary phases (sulfobetaine, phosphocholine, and cysteine). Currently, a variety of neutral, commercially available stationary phases exist, such as Waters XBridge BEH Amide, TSKgel Amide-80 (TOSOH Corporation), and Inertsil HILIC (GL Sciences). Anionic groups include aspartic acid (PolyCAT A™) and carboxylic acid (Shodex VC-50). Cationic groups include amino groups, amines, and imidazoles. TSKgel NH_2_ and HILIC Pak VT-50 are commercially available cationic stationary phase columns. Zwitterionic stationary phases are unique to HILIC, with common commercial examples including sulfobetaine (Atlantis BEH Z-HILIC), phosphocholine(SeQuant^®^ ZIC-cHILIC), and cysteine (Click Xion).

The above classification of HILIC stationary phases based on charge properties provides a framework for understanding their overall diversity. However, for the separation of polar components in TCM, it is more direct and practical to analyze them from the perspective of the surface modifier groups of the stationary phase. In the following section, we will introduce the application of stationary phases in TCM according to the groups used to modify the silica gel surface.

### 2.1 Bare silica stationary phase

The bare silica stationary phase consists of unmodified porous silica gel particles with a surface rich in silanol (silica hydroxyl) groups. In pharmaceutical analysis, most HILIC applications still use underivatized bare silica as the stationary phase. Many column manufacturers have developed silica columns specifically designed for HILIC, such as the Waters Atlantis HILIC Column. In 2010, researchers reported using Waters Atlantis HILIC Silica (150 × 2.1 mm, 3 μm) to isolate the highly polar compound in fenugreek samples (Zhou et al., 2010), achieving the separation of impurities from fenugreek within 4 min. Prior to this, Li et al. established a HILIC/RPLC-TOF MS method and used an Agilent Zorbax RX-SIL column (4.6 × 50 mm, 1.8 μm) to analyze highly polar to weakly polar components of Radix Cyathulae, separating a total of 24 compounds, including carbohydrate compounds, saponins, and others (Ren et al., 2009). Among them, 8 saponins were reported for the first time in this plant, and 4 saponins were proposed to be novel compounds. These results demonstrate that this method has promising application potential.

### 2.2 Zwitterionic stationary phase

Zwitterionics are molecules that contain both positively and negatively charged groups, resulting in a net charge of zero. The zwitterionic HILIC stationary phase is widely used in the analysis of sugars, peptides, drugs, and metabolites. Moreover, it is also commonly employed in the analysis of TCM. Chen et al. utilized a Shiseido PC HILIC column (150 × 2.0 mm, 5 μm) for the separation of the TCM formulation Guanxinning injection (Chen P et al., 2011). In addition, a variety of components in *Salvia miltiorrhiza* were comprehensively analyzed using SeQuant ZIC-cHILIC, a phosphocholine-based zwitterionic stationary phase, which provided good peak shapes and separation performance for phenolic acid analysis (Zhang et al., 2024). Beyond commercially available zwitterionic stationary phases, other customized stationary phases have also been employed in TCM analysis. One of the most commonly used is the cysteine-bonded zwitterionic stationary phase. For example, Li et al. used a self-prepared Click TE-Cys column (150 × 4.6 mm, 5 μm) to analyze toad lactones in toads (Li et al., 2014). Subsequently, they used this stationary phase to separate polar compounds in *Caulis polygoni multiflori* (Liu et al., 2019).

### 2.3 Hydroxyl modified stationary phase

In HILIC, hydroxyl-modified stationary phases represent an important class of chromatographic materials. These materials are modified with hydroxyl (–OH) groups through chemical bonding or physical adsorption. The strong hydrophilicity and hydrogen bonding capabilities of hydroxyl groups are utilized to retain and separate moderately polar compounds. Qin and colleagues used a Phenomenex Luna HILIC column (250 × 4.6 mm, 5 μm), achieving good chromatographic separation of ginsenoside Rb_1_, astragaloside IV, and dulcitol in the TCM oral liquid “*Fufangfufangteng Heji*” (Qin et al., 2011). Cyclodextrins (CDs) are a group of cyclic oligosaccharides, with α-, β-, and γ-CDs classified based on the number of glucose units (Guo et al., 2016; Tian and Liu, 2020; Zhang et al., 2015). With the introduction of the HILIC concept in 1990, β-CD stationary phases began to be applied. Around 2007, Xu’s team developed a β-CD stationary phase via click chemistry, which was later experimentally shown to exhibit both reversed-phase and hydrophilic properties (Xu et al., 2013). This material has also been applied to off-line 2D-RPLC/HILIC and 2D-HILIC/HILIC systems (Liu et al., 2009) to study polar as well as moderately polar compounds in *Carthamus tinctorius Linn*. The results showed that 1879 and 554 peaks could be separated using the two systems, respectively, demonstrating the β-CD phase’s excellent resolution for relatively polar compounds.

### 2.4 Amino-modified stationary phase

Amino-modified columns are stationary phases bonded with amino (–NH_2_) functional groups on a silica gel or polymer matrix. When using this stationary phase, the degree of protonation of the amino group can be controlled by adjusting the pH and ionic strength of the mobile phase, allowing for tunable retention behavior. The amino column has long been considered the gold standard for sugar separation. In previous studies, polyethylenimine (PEI) grafted onto microspheres has been used as a HILIC stationary phase to separate polar compounds such as nucleosides (Peng et al., 2016) and peptides (Chen et al., 2014), although the analysis of oligosaccharides has not been systematically explored. Subsequently, Zhao et al. successfully synthesized two different stationary phase particles—Sil-epoxy-PEI and Sil-chloropropyl-PEI—by grafting PEI onto the surface of silica spheres using two distinct methods (Zhao et al., 2024). It was demonstrated that Sil-epoxy-PEI exhibited relatively high hydrophilicity, and its high retention time and high resolution for standard carbohydrate compounds indicated strong potential for application in carbohydrate separation.

### 2.5 Amide-modified stationary phase

Amide-modified stationary phases typically utilize a matrix of high-purity silica gel or an organic polymer (e.g., polystyrene-divinylbenzene), bonded with amide groups (e.g., acetamide or propionamide) via a silanization reaction. Common commercial amide-modified stationary phases include Sunshell HILIC Amide, XBridge BEH Amide, and Unisol Amide. In the separation and analysis of polar components of TCM, the amide stationary phase is one of the most widely used. Liang et al. employed a 2D liquid chromatography method to separate two fractions of *Scutellaria barbata D. Don* (Liang et al., 2012). Off-line 2D HILIC × HILIC and HILIC × RPLC modes combined with mass spectrometric detection were implemented using XAmide columns (150 × 4.6 mm, 5 μm particle size, Sipore Co., Ltd.) for separation. Using this combined system, a total of 749 peaks were successfully separated and analyzed in *S. barbata D. Don*. Additionally, Liu and colleagues utilized a Waters XBridge Amide Column (4.6 × 150 mm, 3.5 μm) for the analysis of *Cistanche salsa* (Liu et al., 2018) and *Peucedani Radix* in an RPLC-HILIC direct tandem system (Gong et al., 2022). The results demonstrated that the small I.D. RPLC column was suitable as a pre-chromatographic separation column for moderately to highly polar compounds. While relatively low-polarity compounds could not be retained on the RPLC column, they were well retained on the subsequent HILIC column.

## 3 Application of HILIC in the single component analysis of TCM

There are many types of active ingredients in TCM, which can be classified based on their chemical structures, such as polysaccharides, glycosides, alkaloids, organic acids, volatile oils, and terpenes. Among them, polar compounds, such as phenolic acids, alkaloids, and saponins often face technical bottlenecks, including weak retention and insufficient resolution in RPLC. In contrast, HILIC effectively enhances their chromatographic retention behavior through a unique hydrophilic mechanism, significantly improving separation efficiency (Liu et al., 2019). The applications of HILIC in TCM analysis are comprehensively summarized in Table 1.

**TABLE 1:** The application of HILIC in the analysis of TCMs.

Number	Stationary phase type	Mobile phase	Flow rate (mL/min)	Component	TCM	References
1	Click XIon (150 × 4.6 mm, 5 μm)	A: H_2_O B: ACN	1.0	Polysaccharides	*Radix astragali*	Liang et al. (2014)
2	XAmide with neutral amide bonded phase (250 × 4.6 mm, 10 μm)	A: H_2_O B: ACN	1.0	Polysaccharides	*Atractylodes*	Lin et al. (2015)
3	Accucore-150-Amide-HILIC column (100 × 2.1 mm, 2.6 μm)	A: Ammonium acetate B: ACN	0.5	Oligosaccharides	*Radix Rehmanniae*	Wang et al. (2020)
4	TSKgel Amide-80 column (150 × 2.0 mm, 3.0 μm)	A: ACN B: Ammonium acetate	0.2	Nucleosides and nucleobases	*Geosaurus and Leech*	Chen P. et al. (2011)
5	Acquity BEH Amide column (2.1 × 100 mm, 1.7 μm)	A: H_2_O, 10 mM ammonium acetate and 0.8% acetic acid B: ACN and 0.05% acetic acid	0.4	Nucleosides and nucleobases	*Elaphuri Davidiani Cornu and Cervi Cornu*	Li et al. (2013)
6	Acquity UPLC BEH Amide column (2.1 × 100 mm, 1.7 µm)	A: H_2_O, 0.8% acetic acid and 10 mM ammonium acetate B: ACN and 0.1% acetic acid	0.4	Nucleosides and nucleobases	*Animal Horns*	Liu et al. (2015)
7	1D: RP-C18HCE prep column (10 × 250 mm, 10 µm) 2D: HILIC-XAmide prep column (10 × 250 mm, 10 µm)	A: H_2_O and 0.2% v/v formic acid B: 0.2% v/v formic acid methyl alcohol	1D: 3.0 2D: 3.0	Alkaloids	*A. kansuensis*	Cui et al. (2017)
8	ACQUITY UHPLC BEH amide column (2.1 × 100 mm, 1.7 μm)	A: H_2_O and 0.1% formic acid and 10 mM ammonium acetate B: 0.1% formic acid in ACN	0.4	Alkaloids	*Sophora alopecuroides L*	Wang et al. (2018)
9	1D: XBridge Amide column (150 × 4.6 mm, 3.5 μm) 2D: Ultimate amide column (50 × 4.6 mm, 5 μm)	A: H_2_O and 0.1% aqueous formic acid B: ACN	1D: 0.08 2D: 3.0	Flavonoids and alkaloids	*Carthamus tinctorius L*	Wang et al. (2021)
10	1D: Waters Xbridge amide column (4.6 × 150 mm, 3.5 µm) 2D: Waters ACQUITY UPLC BEH C18 column (2.1 × 100 mm, 1.7 µm)	A (1D): H_2_O A (2D): H_2_O and 0.1% formic acid B: ACN	1.0	Gypenosides	*Gynostemma pentaphyllum*	Chen et al. (2023)
11	Analytical/preparative Diol column (250 × 4.6 mm, 10 μm, 250 × 20 mm, 10 μm)	H_2_O and 0.1% formic acid/ACN	1.0 and 19	Iridoid glycosides and flavonoid glycosides	*Hedyotis diffusa*	Dai et al. (2023)
12	1D: Waters XBridge Amide column (4.6 × 150 mm, 3.5 μm) 2D: Waters Acquity BEH C18 column (2.1 × 100 mm, 1.7 μm)	A: H_2_O and 0.1%formic acid B: ACN	1D: 1.0 2D: 0.3	Saponins	*Rhizoma panacis Japonici*	Yasen et al. (2024)
13	1D: Unitary XAmide column (4.6 × 250 mm, 5 μm, Acchrom) 2D: Acquity HSS T3 column (2.1 × 50 mm, 1.7 μm)	A (1D): H_2_O and 0.4% formic acid A (2D): H_2_O and 0.1% formic acid B (1D): ACN and 0.4% formic acid B (2D): ACN and 0.1% formic acid	1D: 1.5 2D: 0.4	Phenolic acids	*Salvia miltiorrhiza Bunge*	Sun et al. (2016)
14	1D: XBridge Amide column (150 × 4.6 mm, 3.5 μm) 2D: Accucore PFP column (50 × 4.6 mm, 2.6 μm)	A (1D): H_2_O and 0.4% formic acid A (2D): H_2_O and 0.1% formic acid B (1D): ACN and 0.4% formic acid B (2D): ACN and 0.1% formic acid	1D: 0.1 2D: 3.0	Phenolic acids	*Salvia miltiorrhiza*	Cao et al. (2018)

ACN: acetonitrile.

### 3.1 Carbohydrates

Polysaccharides, a class of carbohydrate compounds produced from TCM (i.e., *Panax notoginseng*, *Astragalus membranaceus*, *Angelica sinensis*, and *Ganoderma lucidum*), exhibit various bioactivities such as immunomodulatory, antitumor, antioxidant, antiviral, and anti-inflammatory effects (Qiang et al., 2023). They are an important focus in natural medicine research and development. In early 2014, Liang et al. separated polysaccharides from Astragalus using a zwitterionic cysteine-bonded silica column, Click XIon (150 × 4.6 mm, 5 μm) (Liang et al., 2014). Good resolution was achieved in the separation of Astragalus oligosaccharides under HILIC mode. In the same year, Lin and colleagues also applied HILIC to isolate polysaccharides. It was the first time that preparative HILIC using a neutral amide-bonded XAmide column was employed to isolate and identify inulin-type polysaccharides in *Atractylodes*, resulting in the purification of 18 polysaccharides (Lin et al., 2015). Wang et al. developed a method for the simultaneous analysis of Rehmannia mid-ocyclic terpene glycosides and oligosaccharides (Wang et al., 2020). Retention of oligosaccharides on the ACQUITY UPLC BEH HILIC Column and the Accucore-150-Amide-HILIC Column was compared, showing better retention and peak shape on the latter. The method enabled rapid separation of 7 analytes in *Rehmannia* samples.

### 3.2 Nucleosides and nucleobases

Active ingredients in TCM often include nucleosides and nucleobases, found in species such as *Cordyceps sinensis* (Zou et al., 2017) and *P. notoginseng* (Qian et al., 2008), and are considered key quality control markers. Therefore, accurate quantitative and qualitative methods are essential. Chen et al. established a simple HILIC-based method for separating 19 nucleosides and nucleobases from Geosaurus and Leech, with RPLC employed for comparison (Chen X et al., 2011). The compounds were successfully separated using a TSKgel Amide-80 HILIC Column (2.0 × 150 mm, 3 μm). However, many HILIC methods for nucleoside and nucleobase separation still suffer from long analysis times (up to 2 h (Zhao et al., 2011)). To address this, a fast and sensitive ultra-performance HILIC-triple quadrupole mass spectrometry (HILIC-UPLC-TQ-MS/MS) method was developed, using an ACQUITY UPLC BEH Amide column (2.1 × 100 mm, 1.7 μm) with an ACQUITY UPLC BEH Amide VanGuard (1.7 μm) (Li et al., 2013). This method achieved satisfactory separations of 17 polar compounds within 9 min and was applied to determine 14 nucleosides and nucleobases in animal horns (Liu et al., 2015).

### 3.3 Alkaloids

Alkaloids are mainly derived from plants (commonly referred to as “plant alkaloids”), though they are rarely derived from animals. Alkaloids from medicinal plants exhibit antioxidant and anti-inflammatory properties (Zheng et al., 2018). However, some such as quinoline alkaloids are associated with hepatotoxic, neurotoxic, and developmentally toxic effects. Therefore, alkaloid analysis contributes to the quality control of medicinal plants and related pharmaceutical products. Wang et al. developed a method for determining quinolinzine-type alkaloids in *S. alopecuroides* using HILIC-TQ-MS/MS (Wang et al., 2018). Separations were achieved using an ACQUITY UHPLC BEH Amide Column (2.1 × 100 mm, 1.7 μm) and an ACQUITY UHPLC BEH Amide VanGuard pre-column (2.1 × 5 mm, 1.7 μm). Liu and colleagues proposed a 2D RP/HILIC method for targeted separation and purification of oxidized carboline alkaloids and flavonoids, using preparative NP-XAmide and XAmide columns (250 × 4.6 mm, 5 μm) (Cui et al., 2017). Additionally, an on-line integrated HILIC × HILIC system was developed, combining two columns—XBridge amide column (150 × 4.6 mm, 3.5 μm) and Ultimate amide column (5 × 4.6 mm, 5 μm)—for separating flavonoids and alkaloids in safflower (Wang et al., 2021). The 2D system achieved a peak capacity of 81 and higher resolution than the 1D system.

### 3.4 Glycosides

Glycosides are formed by linking sugars or sugar derivatives (e.g., amino sugars, alkyl acids) with non-sugar compounds (aglycones or ligands) through the carbon atoms of the sugar. These abundant glycans give glycosides strong or high polarity properties, posing challenges for conventional HPLC separation. Yasen et al. established an off-line HILIC × RPLC/QTOF-MS system, identifying 307 saponins from *Rhizoma panacis japonica*, 76 of which were reported for the first time (Yasen et al., 2024). A HILIC × RP off-line 2D system was used with a Waters Xbridge Amide column (4.6 × 150 mm, 3.5 μm) for the separation and enrichment of *Gynostemma pentaphyllum* (Chen et al., 2023). A 2D RPLC × HILIC approach was also proposed for isolating iridoid and flavonoid glycosides in *Hedyotis diffusa* (Dai et al., 2023). A preparative Diol column was employed for separation and purification, leading to the identification of 27 compounds.

### 3.5 Phenolic acids

The water-soluble components of *salvia miltiorrhiza* are rich in phenolic acids, which exhibit antioxidant, anti-blood coagulation, and antidiabetic properties (Huang et al., 2012; Zhang et al., 2010), and are widely used in treating cardiovascular, cerebrovascular, and hyperlipidemic diseases (Wang et al., 2007). Therefore, the study of phenolic acids in salvia and related TCMs is valuable for evaluating their medicinal potential. An off-line HILIC × RP-LC/IT-TOF-MS system was developed for determining phenolic acids in *Salvia miltiorrhiza* using a Unitary XAmide column (4.6 × 250 mm, 5 μm, Acchrom) (Sun et al., 2016). An on-line integrated HILIC × RP system was also used for separating polar phenolic acids in salvia, using the XBridge Amide Column (Cao et al., 2018). A total of 196 peaks were well resolved in *Salvia miltiorrhiza*, demonstrating strong selectivity for polar compounds in complex matrices.

## 4 Conclusion

HILIC has emerged as a powerful tool for the separation and analysis of polar compounds in TCM. Over the past decade, various stationary phase modifications have been employed to enhance the separation of polar components. Among them, amide-modified stationary phases are the most commonly selected in the analysis. Furthermore, the application of HILIC in 2D-LC has gradually replaced its standalone use, as its combination with RPLC enables broader separation coverage across diverse TCM components. Nevertheless, the separation of TCM remains a significant challenge due to its complex chemical composition. Although HILIC holds great promise for improving this process, several technical issues remain unresolved. For instance, solvent incompatibility in 2D-LC continues to be a major bottleneck. Additionally, there is a pressing need to develop novel stationary phases that can integrate multiple interaction mechanisms for effective multi-component analysis. In conclusion, advancing the application of HILIC in polar compound analysis of TCM will require collaborative efforts across disciplines to overcome current limitations and expand its analytical capabilities.
